# Magnetic Resonance Imaging as a Readout of CLN5 Gene Therapy Efficacy in Sheep

**DOI:** 10.1002/brb3.70431

**Published:** 2025-04-03

**Authors:** Samantha J. Murray, Mustafa M. Almuqbel, Simon A. Felton, Nickolas J. Palmer, Ashley R. Deane, Daniel J. Myall, Reza Shoorangiz, Arsène Ella, Matthieu Keller, David N. Palmer, Tracy R. Melzer, Nadia L. Mitchell

**Affiliations:** ^1^ Faculty of Agriculture and Life Sciences Lincoln University Canterbury New Zealand; ^2^ Pacific Radiology Group Christchurch New Zealand; ^3^ New Zealand Brain Research Institute Christchurch New Zealand; ^4^ UMR Physiologie de la Reproduction & des Comportements INRAE/CNRS/University of Tours Nouzilly France; ^5^ Department of Radiology University of Otago Christchurch New Zealand; ^6^ Department of Medicine University of Otago Christchurch New Zealand; ^7^ School of Psychology, Speech and Hearing University of Canterbury Christchurch New Zealand

**Keywords:** batten disease, CLN5, gene therapy, intracerebroventricular, intravitreal, magnetic resonance imaging, sheep, therapeutic efficacy

## Abstract

**Purpose:**

Neuronal ceroid lipofuscinoses (NCL; Batten disease) are a group of rare inherited neurodegenerative disorders caused by mutations in one of 13 ceroid lipofuscinosis neuronal (*CLN*) genes. The diseases share a common set of symptoms, including motor and cognitive dysfunction, progressive loss of vision, and seizure activity. A naturally occurring model of CLN5 NCL exists in New Zealand Borderdale sheep, which exhibit similar clinical disease and post‐mortem pathology to the human disease. Recent trials of concurrent intracerebroventricular and intravitreal gene therapy in sheep with CLN5 disease confirmed the therapeutic efficacy of this approach. Given the documented natural history of brain volume changes, detected by MRI, in sheep with CLN5 disease, the current study sought to utilize MRI as both a longitudinal readout and cross‐sectional measure of therapeutic efficacy in treated sheep.

**Method:**

Sheep treated at a pre‐symptomatic timepoint underwent five T1‐weighted structural MRI scans between 5 and 18 months of age. Sheep treated at early and advanced symptomatic disease stages underwent a single MRI at 18 months of age. All scans from treated sheep were compared to historical healthy control and affected untreated sheep at each age.

**Finding:**

Pre‐symptomatic treated sheep showed growth in intracranial volume at a comparable rate to healthy control sheep over the course of the study. Whilst grey matter volume decreased and cerebrospinal fluid volume increased in treated sheep, this was to a much smaller degree than in untreated affected sheep. The majority of the cortical regions assessed showed stable volumes over the course of the study, with the notable exception of the cerebellum.

Both early and advanced symptomatic treated sheep showed intracranial volumes comparable to untreated affected sheep at 18 months of age. However, when individual tissue types were assessed, grey and white matter were significantly larger, and cerebrospinal fluid was significantly smaller in early symptomatic sheep compared to untreated affected sheep, while the same volumes in advanced symptomatic treated sheep were comparable to untreated affected sheep. Cortical regions assessed showed an age‐at‐treatment and dose effect.

**Conclusion:**

This study has demonstrated that MRI, a clinically relevant outcome measure, can be successfully utilized to assess therapeutic efficacy in a large animal model of CLN5 NCL, both in a longitudinal study and a cross‐sectional study when robust natural history data is available for comparison.

## Graphical abstract

Sheep with CLN5 Batten disease that received combination intracerebroventricular and intravitreal gene therapy underwent MRI scanning to assess global and regional brain volumes and determine the value of MRI to detect therapeutic efficacy. Sheep that were treated pre‐symptomatically underwent longitudinal scanning between 5 and 18 months of age, while sheep treated at early and advanced disease stages underwent a single MRI at 18 months of age. Pre‐symptomatic treated sheep showed growth in intracranial volume at a comparable rate to healthy control sheep over the course of the study. Cortical regions showed stable volumes over time in treated sheep compared to untreated affected sheep. In early‐ and advanced‐symptomatic treated sheep assessed at 18 months of age there was an age‐at‐treatment and dose effect on global and regional brain volumes.

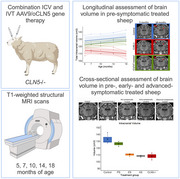

## 1 Introduction

Neuronal ceroid lipofuscinoses (NCL), also known as Batten disease, are a group of fatal recessively inherited neurodegenerative diseases that primarily affect children, caused by mutations in one of 13 *CLN* genes (*CLN1‐8* and *CLN10‐14*) and characterized by the accumulation of lysosomal storage material in cells and widespread brain atrophy (Mole and Cotman 2015). Common symptoms across all variants of NCL include learning and memory deficits, seizures, and progressive loss of vision and motor skills resulting in premature death (Haltia 2003). Currently, the only approved disease‐modifying therapeutic for NCL is an enzyme replacement therapy for CLN2 disease; however, there are several promising candidate therapies currently in clinical trials for several forms of NCL (Liu et al. 2020; Schulz et al. 2018, 2024).

NCLs occur naturally in many species aside from humans, including mice, dogs, cattle, sheep, and non‐human primates. New Zealand Borderdale sheep with CLN5 disease represent one of the more well‐studied large animal models of naturally occurring NCL (Frugier et al. 2008; Jolly et al. 2002; Mitchell et al. 2018). Affected Borderdale (*CLN5^−/−^*) sheep exhibit lysosomal storage, brain atrophy, progressive loss of vision, and motor and cognitive decline and die prematurely between 18 and 24 months of age (Mitchell, Russell, et al. 2023).

Viral‐mediated gene replacement therapy is a promising potential therapeutic strategy for treating NCL. Sheep with CLN5 disease treated with intracerebroventricular (ICV) gene therapy prior to symptom onset maintain brain volume and survive up to triple their normal lifespan (Mitchell, Murray, et al. 2023). More recent trials have focused on treating symptomatic sheep using a dual route of administration targeting both the brain (ICV) and eye (intravitreal; IVT). These trials have shown retention of cognition and vision in treated sheep up to a pre‐determined end‐point of 24 months of age, long after the development of profound blindness in untreated affected sheep (Murray, Wellby, et al. 2023).

Magnetic resonance imaging (MRI) is a technique commonly used in clinical settings to monitor disease progression or assess therapeutic efficacy in a range of neurodegenerative diseases. A recent study used MRI to monitor intracranial volumes as well as grey matter (GM), white matter (WM), and cerebrospinal fluid (CSF) volumes in sheep with CLN5 disease compared to healthy control sheep between 5 and 18 months of age (Murray, Almuqbel, et al. 2023). Affected sheep displayed a gradual decline in intracranial volumes over time, which was primarily driven by a decline in GM volumes and a concurrent increase in CSF volumes. In contrast, healthy control sheep brains grew in volume over the same time. The study also found a significant decline in the volumes of all cortical regions assessed in CLN5‐affected sheep and a relative sparing of subcortical regions (Murray, Almuqbel, et al. 2023). These data establish MRI as a useful technique for tracking brain volume changes in vivo in sheep with CLN5 Batten disease.

The current study aimed to use MRI to assess the therapeutic efficacy of a dual route of administration of self‐complementary adeno‐associated virus expressing ovine *CLN5* (scAAV9/oCLN5) in sheep with CLN5 Batten disease (Murray, Wellby, et al. 2023). Sheep treated at a pre‐symptomatic time point were enrolled in a longitudinal MRI study consisting of scans between 5 and 18 months of age to align with the previous study. A cross‐sectional study was also performed for sheep treated with scAAV9/oCLN5 at early and advanced symptomatic disease stages. These animals were scanned once at 18 months of age and their MRI data compared to age‐matched pre‐symptomatic treated, healthy control, and untreated sheep.

## 2 Materials and Methods

### 2.1 Animals, Vector, and Gene Therapy

The 16 animals included in this study have previously been described (Murray, Almuqbel, et al. 2023, Murray, Wellby, et al. 2023). In brief, Borderdale sheep were genetically diagnosed at birth as clinically healthy CLN5 heterozygous (CLN5^+/−^) or CLN5 affected (CLN5^−/−^) as previously described (Frugier et al. 2008). They were maintained at Lincoln University under NIH guidelines for the Care and Use of Animals in Research and the NZ Animal Welfare Act (1999). All experimental protocols were approved by the Lincoln University Animal Ethics and Institutional Biosafety Committees.

Nine CLN5^−^ *^/−^* ewes received combination bilateral ICV and unilateral IVT administration of a recombinant self‐complementary adeno‐associated virus serotype 9 (AAV9) encoding a codon‐optimized ovine *CLN5* transgene (oCLN5opt) driven by the chicken beta actin (CBh) promoter (scAAV9/oCLN5) at three different disease stages (pre‐symptomatic; 3 months, early symptomatic; 6 months, or advanced symptomatic; 9 months) as previously described (Murray, Wellby, et al. 2023). Sheep treated at the pre‐symptomatic time point received a moderate ICV dose (2.9 × 10^11^ viral genomes (vg)), while the early and advanced symptomatic treated animals received a high ICV dose (3.3 × 10^12^ vg). All groups received the same IVT dose of 6.5 × 10^10^ vg (Murray, Wellby, et al. 2023).

Control data were from untreated clinically normal (CLN5^+/−^, *n* = 3) and affected (CLN5^−/−^, *n* = 4) ewes.

### 2.2 Clinical Scoring

Monthly neurological clinical assessments were performed on the treated CLN5^−/−^ sheep and age‐matched cohorts of healthy CLN5^+/−^ and untreated CLN5^−/−^ sheep by two independent blinded investigators using the Ovine Batten Disease Rating Scale (oBDRS) (Mitchell et al. 2018). oBDRS data from these sheep has been previously published (Murray, Wellby, et al. 2023).

### 2.3 MRI Scanning and Image Processing

Scanning and processing methods have been previously described (Murray, Almuqbel, et al. 2023). MRI scans were performed on pre‐symptomatic ICV/IVT‐treated sheep and concurrent healthy CLN5^+/−^ and untreated CLN5^−/−^ controls at 5, 7, 10, 14, and 18 months of age. Early‐ and advanced symptomatic ICV/IVT‐treated sheep received a single MRI scan at 18 months of age. Sheep were anesthetized with intravenous administration of 0.5 mg/kg diazepam and 10 mg/kg ketamine, intubated, and maintained on isoflurane gas inhalation (1.5%–3% v/v to effect in oxygen). They were scanned in the right lateral recumbent position in a Siemens 3‐Tesla MAGNETOM Skyra MRI scanner (Siemens Healthcare, Erlangen, Germany) with the 20‐channel head coil. Structural T1‐weighted imaging was performed to measure tissue volumes using the following parameters: T1‐weighted (MPRAGE; magnetization‐prepared rapid acquisition with gradient echo), TE/TR = 2.02/2500 ms, TI = 900 ms, flip angle = 12°, FOV = 192 mm, slice thickness = 0.75 mm, matrix = 256 × 256, voxel size = 0.75 × 0.75 × 0.75 mm^3^, NEX = 4, scan time = 25:32, slice acceleration, GRAPPA = 2. T1 images for each sheep were segmented using SPM8 (Statistical Parametric Mapping, UCL Queen Square Institute of Neurology, London, UK) (http://www.fil.ion.ucl.ac.uk/spm) with Borderdale‐specific ovine tissue probability maps (Murray, Almuqbel, et al. 2023) generated based on published methods (Ella et al. 2017), producing GM, WM, and CSF maps at each age. Intracranial volumes were calculated at each age as the sum of the GM, WM, and CSF volumes. The volumes of 12 regions of interest (lateral ventricles, primary motor, sensory, and visual cortices, frontal and parieto‐occipital cortices, cerebellum, caudate nucleus, putamen, and thalamus) selected a priori and defined with a sheep‐specific atlas and parcellations (Ella et al. 2017) were also extracted. Three‐dimensional lateral ventricle volumetrics were performed using the 3D Slicer 4.11 freeware (http://www.slicer.org) (Fedorov et al. 2012; Russell et al. 2018).

### 2.4 Statistical Analysis

For the longitudinal study, brain volumes were compared across time and groups using linear mixed effects regression models with lme4 in R v4.0.5 (Bates et al. 2015; R Core Team 2021). A regression model was fit for each tissue type (GM, WM, and CSF volumes) and each region of interest (lateral ventricles, primary motor, sensory, and visual cortices, frontal and parieto‐occipital cortices, cerebellum, caudate nucleus, putamen, and thalamus) to determine the effects of group, time, and group‐by‐time interactions on volume. A varying intercept was included per sheep. Sheep‐level predictors were group (control, affected, treated); measurement‐level predictors included intracranial volume and time from baseline (age), with appropriate interactions with groups. Tukey HSD (Honest Significant Differences) was used to perform multiple pairwise comparisons to assess significant differences between control, affected, and treated groups while accounting for age. A 7‐month scan in one CLN5*^−/−^* animal was excluded from analysis as the scan images showed severe movement artifact, making volume extraction unreliable and inaccurate.

For the cross‐sectional study (at the 18‐month timepoint), a one‐way ANOVA was performed using the car package in R (Fox and Weisberg 2011) to assess differences in volume between control, untreated CLN5*^−^* ^/^ *^−^*, and the three treatment groups (pre‐, early, and advanced symptomatic treated) for each tissue type (intracranial volume, GM, WM, and CSF) and region of interest (lateral ventricles, primary motor, sensory, and visual cortices, frontal and parieto‐occipital cortices, and the caudate nucleus. Assumptions of the ANOVA were tested using Levene's test for homogeneity of variance and Shapiro–Wilk test for normal distribution. Tukey HSD (Honest Significant Differences) was used to perform multiple pairwise comparisons to assess significant differences between each treatment group and controls and between each treatment group and untreated CLN5*^−^* ^/^ *^−^* sheep.

The Pearson correlation coefficient was used to assess the linear relationship between MRI‐derived cortical volumes and post‐mortem cortical thickness. Analysis was performed in GraphPad Prism (Version 10.3.1). The results are reported as the Pearson correlation coefficient (*r*) and the associated *p* value.

## 3 Results

### 3.1 Previously Published In‐Life Efficacy Endpoints

A summary of the in‐life efficacy endpoints for the 24‐month treatment study, previously reported in Murray, Wellby, et al. 2023, is presented in Table 1. One sheep treated pre‐symptomatically with a moderate scAAV9/oCLN5 dose (1124/18) remained clinically stable for the duration of the study, while the remaining two sheep in this cohort showed mild clinical decline. All sheep in the early symptomatic high‐dose treated group and two in the advanced symptomatic high‐dose treated group remained clinically stable (Table 1, Murray, Wellby, et al. 2023).

**TABLE 1:** Summary of in‐life efficacy end‐points following ICV/IVT scAAV9.oCLN5 treatment.

Treatment groups	Treatment	ICV dose (vg)	IVT dose (vg)	Sheep	Treatment age (months)	Endpoint (months)	Clinical description	Rate of decline^*^
Pre‐ symptomatic	ICV/IVT scAAV9/oCLN5	MD 2.9 × 10^11^	6.5 × 10^10^	1102/18	3.6	24.3	Mild decline	−0.6
				1124/18	3.3	24.0	Clinically stable	−0.2
				1128/18	3.3	24.0	Mild decline	−0.6
Early‐ symptomatic	ICV/IVT scAAV9/oCLN5	HD 3.3 × 10^12^	6.5 × 10^10^	1151/18	6.3	24.0	Clinically stable	0
				1154/18	6.3	24.0	Clinically stable	0
				1157/18	6.1	23.8	Clinically stable	0.1
Advanced‐symptomatic	ICV/IVT scAAV9/oCLN5	HD 3.3 × 10^12^	6.5 × 10^10^	1131/18	9.5	24.1	Mild decline	0
				1138/18	9.4	24.0	Clinically stable	0
				1170/18	8.9	23.5	Clinically stable	0.1
Control CLN5± (*n* = 12)	Nil	N/A	N/A	N/A	N/A	>24	Normal	0.0
Untreated CLN5*^−^* ^/^ *^−^* (*n* = 15)	Nil	N/A	N/A	N/A	N/A	18.6	Deceased, advanced clinical disease	−1.7

*Note*: For treated sheep this is from baseline (treatment age) to current age; for controls this is from 3 months to current age. Adapted from Murray, Wellby, et al. 2023.

Abbreviations: HD high dose; ICV intracerebroventricular; m months; MD moderate dose; N/A not applicable; oBDRS ovine Batten disease rating scale; viral genomes.

^*^Rate of decline in oBDRS units per month.

### 3.2 Longitudinal MRI of Pre‐Symptomatic scAAV9/oCLN5 Treated Sheep

**FIGURE 1:**
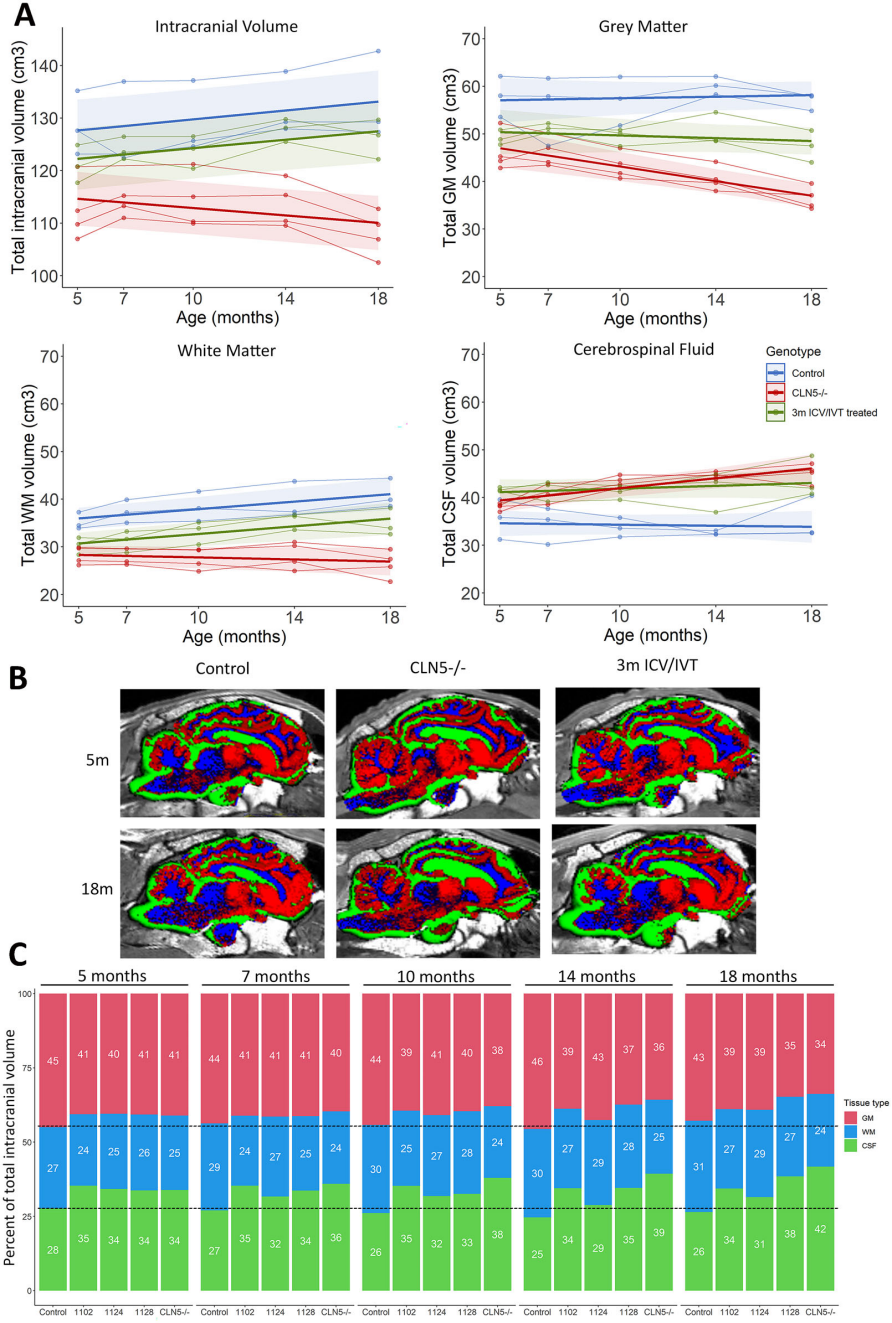
Longitudinal MRI brain changes following pre‐symptomatic ICV/IVT treatment with scAAV9/oCLN5. (A) Total MRI‐derived intracranial, grey matter (GM), white matter (WM), and cerebrospinal fluid (CSF) volumes of pre‐symptomatic ICV/IVT treated sheep (*n* = 3) compared to concurrent healthy control CLN5^+/−^ (*n* = 3) and untreated CLN5^−/−^ (*n* = 4) sheep. Individual animals are represented by data points connected by thin lines while thick lines indicate the linear regression line for each experimental group. Shaded areas indicate 95% confidence intervals of the regression model. (B) Segmented GM (red), WM (blue), and CSF (green) in treated, control, and *CLN5* ^−/−^ sheep superimposed over T1‐weighted images in the sagittal plane at baseline (5 m) and 18 m of age. **C**. Proportions of GM, WM, and CSF at each age in individual treated sheep (1102, 1124, 1128) compared to average healthy control CLN5± (*n* = 3) and average untreated *CLN5* ^−/−^ (*n* = 4) sheep. Dashed lines indicate control values at baseline.

Longitudinal MRI scanning of the pre‐symptomatic moderate‐dose treated sheep showed a trend towards a gradual increase in intracranial volume over time at the same rate as control CLN5^+/−^ sheep (0.4 cm^3^/month), while untreated CLN5^−/−^ animals showed a decline in intracranial volume of 0.4 cm^3^/month (Figure 1A, Table 2). However, two pre‐symptomatic treated sheep (1102/18 and 1128/18) did show decreasing intracranial volume between 14 and 18 months of age. On average, pre‐symptomatic treated sheep lost 1.7 mL of GM and gained 4.6 mL of WM from baseline to 18 months, which is in stark contrast to untreated CLN5^−/−^ animals who lost an average of 9.7 mL and 1.9 mL of grey and WM, respectively, over the same period (Figure 1A). Total CSF volume, which typically increases with brain atrophy, remained relatively stable in control CLN5^+/−^ animals (0.06 cm^3^/month), but increased dramatically by an average of 7.7 cm^3^ (0.5 cm^3^/month) in untreated CLN5^−/−^ animals. Whilst CSF volumes in treated animals were already elevated at baseline, they had only increased by an average of 2.2 cm^3^ (0.1 cm^3^/month) at the 18‐month scan (Figure 1A, Table 3). Volumes of GM, WM, and CSF were expressed as a proportion of total intracranial volume for each treated animal compared to controls to visualize relative changes in tissue types over time (Figure 1C). The proportions of GM, WM, and CSF remained relatively stable in control CLN5^+/−^ sheep over time, while in untreated CLN5^−/−^ sheep the proportion of GM decreased as the proportion of CSF increased. Pre‐symptomatic treated sheep had proportions of GM, WM, and CSF comparable to untreated CLN5^−/−^ sheep at 5 months of age. In two of the three treated sheep (1102/18 and 1128/18), GM began to decline from 10 months of age with a concurrent increase in the proportion of CSF, while the third sheep (1124/18) showed a similar change in proportions between 14 and 18 months of age. At 18 months of age, the proportions of GM, WM, and CSF in pre‐symptomatic treated sheep sat between values for control CLN5^+/−^ and untreated CLN5^−/−^ sheep, with treated animal 1124/18 being the most comparable to control CLN5^+/−^ sheep (Figure 1C).

**TABLE 2:** Linear mixed effects regression model comparing rate of change in volume between control, CLN5 affected, and CLN5 treated sheep.

ICV	CLN5^+/−^	CLN5^−/−^	3 m ICV/IVT treated
Volume at 5 m (cm^3^)	129 [114, 144]	112 [103, 122]	121 [112, 130]
Volume at 18 m (cm^3^)	133 [112, 154]	108 [101, 115]	126 [117, 136]
Slope (cm^3^/month)	0.4 [0.1, 0.7]	−0.4 [−0.6, −0.1]	0.4 [0.1, 0.7]
**GM**	**CLN5^+/−^**	**CLN5^−/−^**	**3 m ICV/IVT treated**
Volume at 5 m (cm^3^)	58 [47, 58]	46 [39, 53]	49 [45, 49]
Volume at 18 m (cm^3^)	57 [52, 61]	36 [33, 40]	47 [39, 56]
Slope (cm^3^/month)	0.09 [−0.03, 1.3]	−0.8 [−1, −0.4]	−0.1 [−0.5, 0.3]
**WM**	**CLN5^+/−^**	**CLN5^−/−^**	**3 m ICV/IVT treated**
Volume at 5 m (cm^3^)	35 [31, 40]	28 [25, 31]	30 [26, 35]
Volume at 18 m (cm^3^)	41 [33, 49]	26 [22, 31]	35 [28, 42]
Slope (cm^3^/month)	0.4 [0.2, 0.6]	−0.1 [−0.3, 0.05]	0.4 [0.2, 0.6]
**CSF**	**CLN5^+/−^**	**CLN5^−/−^**	**3 m ICV/IVT treated**
Volume at 5 m (cm^3^)	36 [25, 46]	38 [37, 29]	42 [41, 43]
Volume at 18 m (cm^3^)	35 [24, 46]	45 [42, 48]	44 [33, 55]
Slope (cm^3^/month)	−0.06 [−0.5, 0.4]	0.5 [0.2, 0.9]	0.1 [−0.3, 0.6]

*Note*: Numbers in square brackets indicate 95% confidence intervals. The slope is considered significantly different from zero if the 95% confidence interval does not include zero. Only the first (5 m) and last (18 m) individual time points are displayed, but the slope is the model output that was estimated using five time points (5, 7, 10, 14, and 18 months).

**TABLE 3:** Linear mixed effects regression model comparing rate of change in volume between control and CLN5 affected sheep.

Primary motor cortex	CLN5^+/−^	CLN5^−/−^	3 m ICV/IVT treated
Volume at 5 m (cm^3^)	1 [0.9, 1.1]	0.8 [0.7, 1]	0.9 [0.8, 1.0]
Volume at 18 m (cm^3^)	1 [0.9, 1.1]	0.6 [0.6, 0.7]	0.9 [0.8, 1.0]
Slope (cm^3^/month)	0.003 [−0.004, 0.01]	−0.02 [−0.02, −0.01]	0.003 [−0.004, 0.009]
**Primary sensory cortex**	**CLN5^+/−^**	**CLN5^−/−^**	**3 m ICV/IVT treated**
Volume at 5 m (cm^3^)	1.4 [1.1, 1.6]	1.1 [0.9, 1.3]	1.3 [1.1, 1.5]
Volume at 18 m (cm^3^)	1.3 [1, 1.5]	0.7 [0.6, 0.8]	1.3 [1.1, 1.5]
Slope (cm^3^/month)	−0.004 [−0.01, 0.004]	−0.03 [−0.04, −0.03]	−0.0005 [−0.008, 0.008]
**Parieto‐occipital cortex**	**CLN5^+/−^**	**CLN5^−/−^**	**3 m ICV/IVT treated**
Volume at 5 m (cm^3^)	2 [1.3, 2.6]	1.3 [1, 1.7]	1.5 [1.3, 1.8]
Volume at 18 m (cm^3^)	1.8 [1.6, 2]	0.8 [0.6, 0.9]	1.3 [1.1, 1.5]
Slope (cm^3^/month)	−0.004 [−0.02, 0.01]	−0.04 [−0.06, −0.03]	−0.02 [−0.03, 0.003]
**Orbital gyrus**	**CLN5^+/−^**	**CLN5^−/−^**	**3 m ICV/IVT treated**
Volume at 5 m (cm^3^)	0.9 [0.8, 1]	0.8 [0.7, 0.8]	0.8 [0.7, 0.9]
Volume at 18 m (cm^3^)	0.8 [0.7, 0.9]	0.6 [0.5, 0.6]	0.8 [0.6, 1.0]
Slope (cm^3^/month)	−0.003 [−0.01, 0.005]	−0.02 [−0.02, −0.01]	−0.0003 [−0.008, 0.008]
**Orbitofrontal gyrus**	**CLN5^+/−^**	**CLN5^−/−^**	**3 m ICV/IVT treated**
Volume at 5 m (cm^3^)	1.8 [1.7, 1.9]	1.5 [1.2, 1.9]	1.6 [1.3, 1.9]
Volume at 18 m (cm^3^)	1.7 [1.6, 1.8]	1 [0.8, 1.1]	1.5 [1.0, 2.1]
Slope (cm^3^/month)	−0.003 [−0.02, 0.02]	−0.05 [−0.06, −0.03]	−0.006 [−0.03, 0.01]
**Cerebellum**	**CLN5^+/−^**	**CLN5^−/−^**	**3 m ICV/IVT treated**
Volume at 5 m (cm^3^)	5.4 [4.7, 6.0]	5.1 [4.4, 5.8]	5.4 [4.5, 6.2]
Volume at 18 m (cm^3^)	5.1 [4.6, 5.5]	5.8 [5.1, 6.5]	5.6 [5.0, 6.1]
Slope (cm^3^/month)	−0.02 [−0.04, 0.01]	0.05 [0.02, 0.07]	0.01 [−0.01, 0.04]
**Entolateral gyrus**	**CLN5^+/−^**	**CLN5^−/−^**	**3 m ICV/IVT treated**
Volume at 5 m (cm^3^)	0.7 [0.5, 0.9]	0.6 [0.5, 0.6]	0.6 [0.5, 0.6]
Volume at 18 m (cm^3^)	0.7 [0.6, 0.7]	0.4 [0.3, 0.4]	0.5 [0.5, 0.5]
Slope (cm^3^/month)	−0.002 [−0.007, 0.005]	−0.02 [−0.02, −0.01]	−0.003 [−0.009, 0.003]
**Lateral gyrus**	**CLN5^+/−^**	**CLN5^−/−^**	**3 m ICV/IVT treated**
Volume at 5 m (cm^3^)	1.6 [1.2, 2.1]	1 [0.9, 1.2]	1.1 [0.9, 1.3]
Volume at 18 m (cm^3^)	1.5 [1.2, 1.8]	0.7 [0.6, 0.9]	1.1 [0.8, 1.3]
Slope (cm^3^/month)	−0.002 [−0.01, 0.008]	−0.02 [−0.03, −0.01]	−0.006 [−0.02, 0.003]
**Occipital lobe**	**CLN5^+/−^**	**CLN5^−/−^**	**3 m ICV/IVT treated**
Volume at 5 m (cm^3^)	1.4 [1.1, 1.7]	1.1 [1, 1.3]	1.1 [1.0, 1.1]
Volume at 18 m (cm^3^)	1.4 [1.2, 1.6]	0.8 [0.7, 0.9]	0.9 [0.3, 1.5]
Slope (cm^3^/month)	−0.0006 [−0.02, 0.02]	−0.03 [−0.04, −0.01]	−0.02 [−0.04, −0.003]
**Caudate nucleus**	**CLN5^+/−^**	**CLN5^−/−^**	**3 m ICV/IVT treated**
Volume at 5 m (cm^3^)	1.6 [1.5, 1.7]	1.5 [1.4, 1.7]	1.6 [1.5, 1.7]
Volume at 18 m (cm^3^)	1.6 [1.5, 1.8]	1.3 [1.3, 1.4]	1.6 [1.6, 1.7]
Slope (cm^3^/month)	0.005 [−0.002, 0.01]	−0.02 [−0.02, −0.01]	0.003 [−0.004, 0.01]
**Putamen**	**CLN5^+/−^**	**CLN5^−/−^**	**3 m ICV/IVT treated**
Volume at 5 m (cm^3^)	0.9 [0.9, 1]	0.9 [0.7, 1.1]	0.9 [0.8, 1.0]
Volume at 18 m (cm^3^)	0.8 [0.6, 0.9]	0.7 [0.8, 1]	0.9 [0.8, 1.0]
Slope (cm^3^/month)	−0.007 [−0.01, 0.0009]	−0.004 [−0.01, 0.002]	−0.003 [−0.009, 0.004]
**Thalamus**	**CLN5^+/−^**	**CLN5^−/−^**	**3 m ICV/IVT treated**
Volume at 5 m (cm^3^)	1.8 [1.6, 2.1]	1.8 [1.6, 2.1]	1.9 [1.8, 2.1]
Volume at 18 m (cm^3^)	1.9 [1.8, 2.1]	1.8 [1.6, 2]	2.0 [1.7, 2.3]
Slope (cm^3^/month)	0.009 [0.001, 0.02]	−0.003 [−0.009, 0.004]	0.002 [−0.005, 0.01]

*Note*: Numbers in square brackets indicate 95% confidence intervals. The slope is considered significantly different from zero if the 95% confidence interval does not include zero. Only the first (5 m) and last (18 m) individual time points are displayed, but the slope is the model output that was estimated using five time points (5, 7, 10, 14, and 18 months).

**FIGURE 2:**
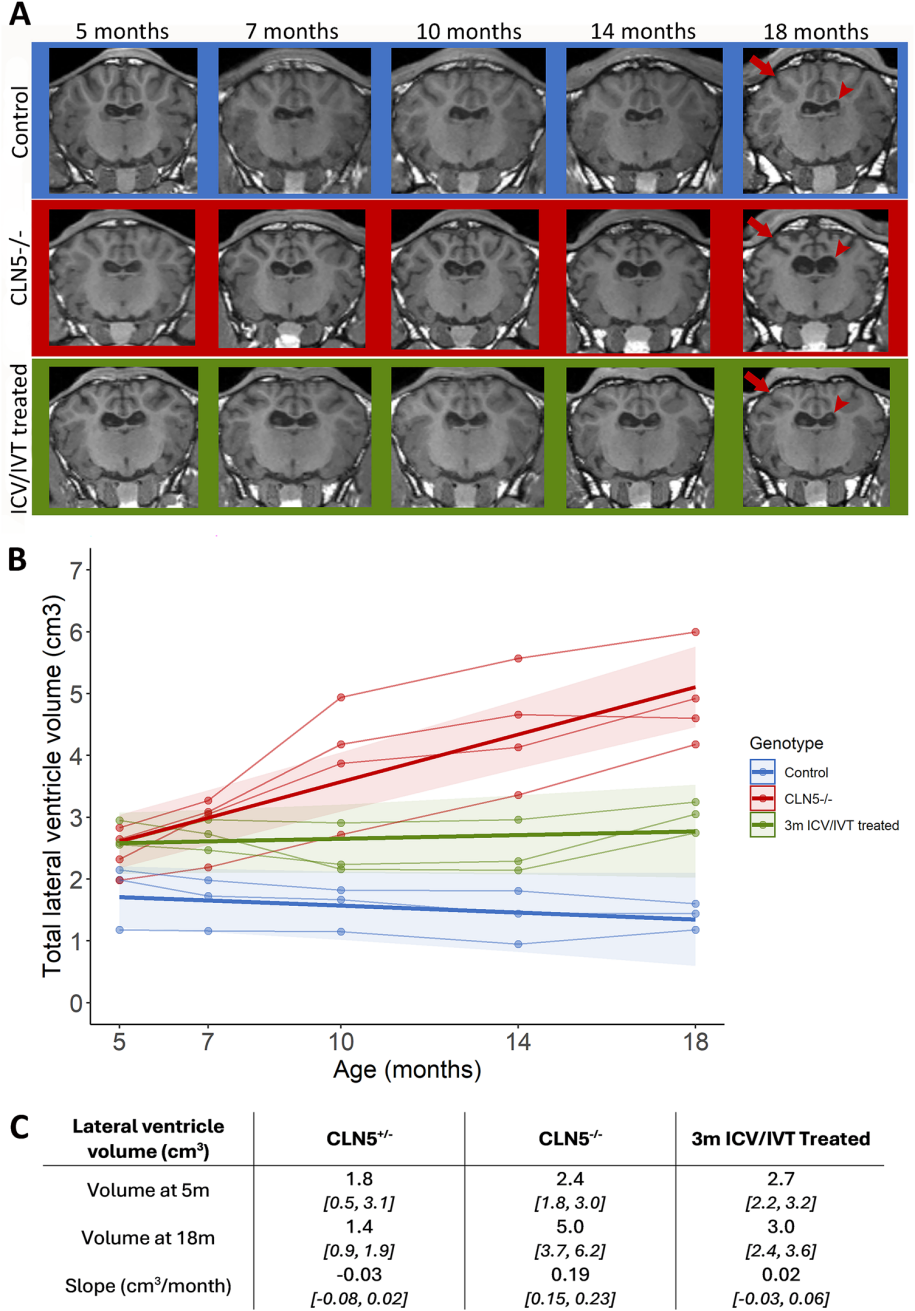
Longitudinal lateral ventricle volume changes following pre‐symptomatic ICV/IVT treatment with scAAV9/oCLN5. (A) Representative longitudinal T1‐weighted coronal MR brain images of a healthy control CLN5^+/−^ (blue panel), untreated CLN5^−/−^ (red panel) and ICV/IVT treated pre‐symptomatic sheep (green panel). The cerebral cortex (red arrow) and lateral ventricles (red arrowhead) are indicated. (B) Total MRI‐derived lateral ventricular volume of pre‐symptomatic ICV/IVT treated sheep (*n* = 3) compared to concurrent healthy control *CLN5^+/−^* (*n* = 3) and untreated *CLN5^−/−^* (*n* = 4) sheep. Individual animals are represented by data points connected by thin lines while thick lines indicate the linear regression line for each experimental group. Shaded areas indicate 95% confidence intervals of the regression model. (C) Comparison of volumes at baseline (5 m) and 18 m and the slope of change. Numbers in square brackets indicate the 95% confidence intervals.

In scan images, widening of the lateral ventricles and cortical atrophy were evident at 18 months of age in untreated CLN5^−/−^ sheep; however, the same was not observed in pre‐symptomatic treated sheep (Figure 2A). Lateral ventricle volume was quantified over time in pre‐symptomatic treated sheep and compared to CLN5^±^ and CLN5^−/−^ controls. Whilst ventricles of pre‐symptomatic treated animals were enlarged compared to control CLN5^+/−^ sheep at the baseline scan, there was only a small average increase over time (0.02 cm^3^/month, Figure 2). In contrast, ventricular volume in untreated CLN5^−/^ *^−^* sheep increased at a rate of 0.19 cm^3^/month (Figure 2).

**FIGURE 3:**
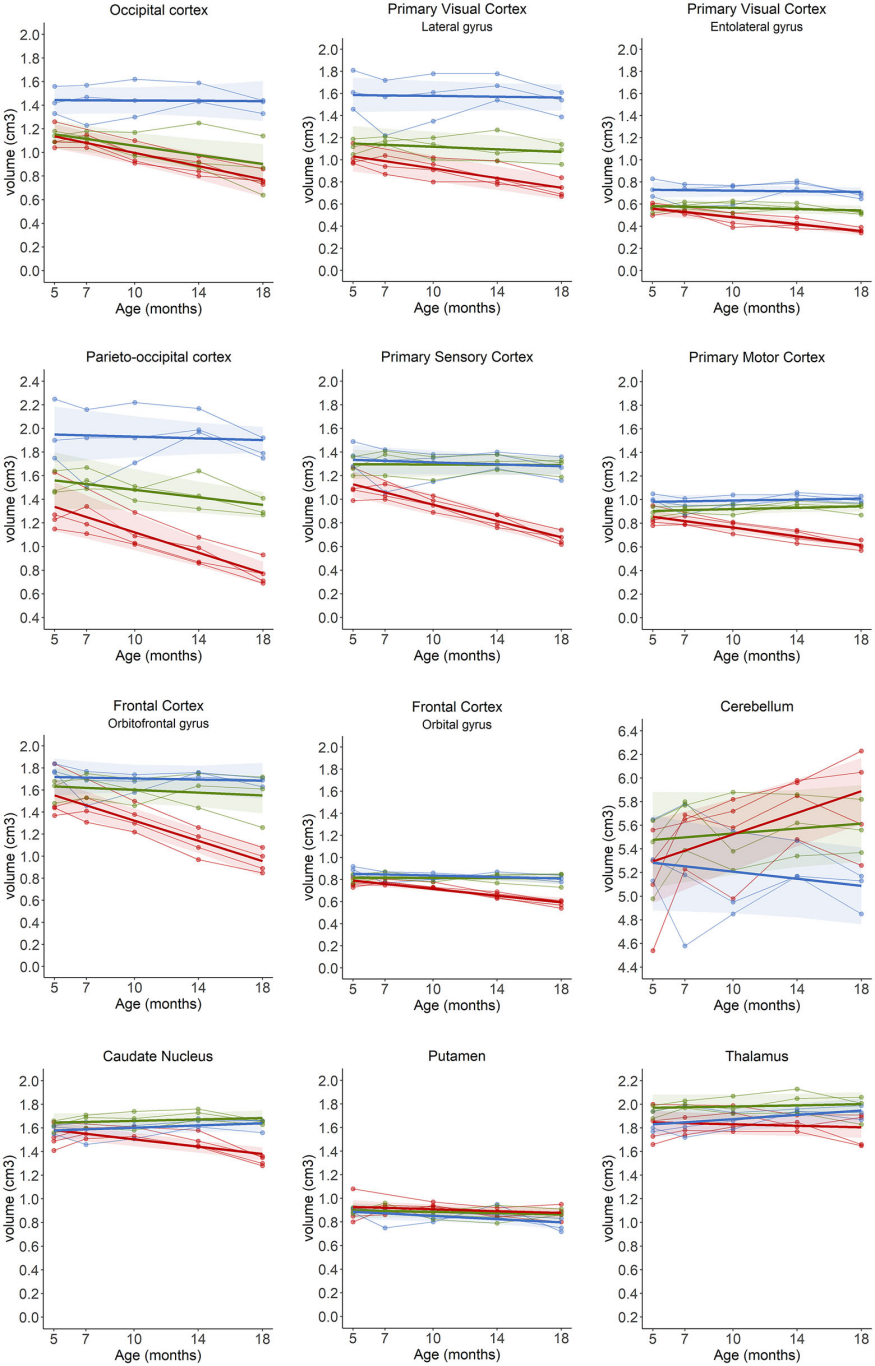
Regional brain volume changes following pre‐symptomatic ICV/IVT treatment with scAAV9/oCLN5. Individual animals are represented by data points connected by thin lines while thick lines indicate the linear regression line for each experimental group. Shaded areas indicate 95% confidence intervals of the regression model.

Several cortical regions of interest were also assessed for volume changes over time using published sheep MRI atlases (Ella et al. 2017; Ella and Keller 2015). Of particular interest were those regions identified in previous systematic histological studies to degenerate the most in ovine NCL (Mitchell, Russell, et al. 2023), including the occipital cortex, primary visual cortices (lateral gyrus and entolateral gyrus), parieto‐occipital cortex, primary sensory cortex, primary motor cortex, frontal cortex (orbital gyrus and orbitofrontal gyrus), and cerebellum. As previously published, all regions with the exception of the cerebellum progressively declined in volume from baseline to the 18‐month scan in untreated CLN5^−/−^ sheep (Murray, Almuqbel, et al. 2023), while there was no change in volume in these regions in control CLN5^+/−^ sheep (Figure 3, Table 3). However, on average in pre‐symptomatic treated sheep, there was no significant change in the volume of the primary visual, motor, sensory, frontal, or parieto‐occipital cortices from baseline to 18 months of age (Figure 3, Table 3). There was a decline in the average volume of the treated parieto‐occipital cortex of 0.02 cm^3^/month; however, this was half the rate of decline of untreated CLN5^−/−^ sheep (0.04 cm^3^/month). There was a significant decline in the volume of the occipital cortex, which was in line with untreated CLN5^−/−^ sheep (0.02 cm^3^/month and 0.03 cm^3^/month, respectively, Figure 3, Table 3).

Volume changes in subcortical regions (caudate nucleus, putamen, and thalamus) were also assessed between baseline and 18 months of age. There was no difference in the volume of the putamen and thalamus between groups over time. The caudate nucleus showed a significant decline in volume of 0.02 cm^3^/month in untreated CLN5^−/−^ sheep; however, there was no significant change in volume in control CLN5^+/−^ or pre‐symptomatic treated sheep (Figure 3, Table 3).

### 3.3 Cross‐Sectional Comparison of Pre‐, Early and Advanced Symptomatic scAAV9/oCLN5 Treated Sheep at 18 Months of Age

**FIGURE 4:**
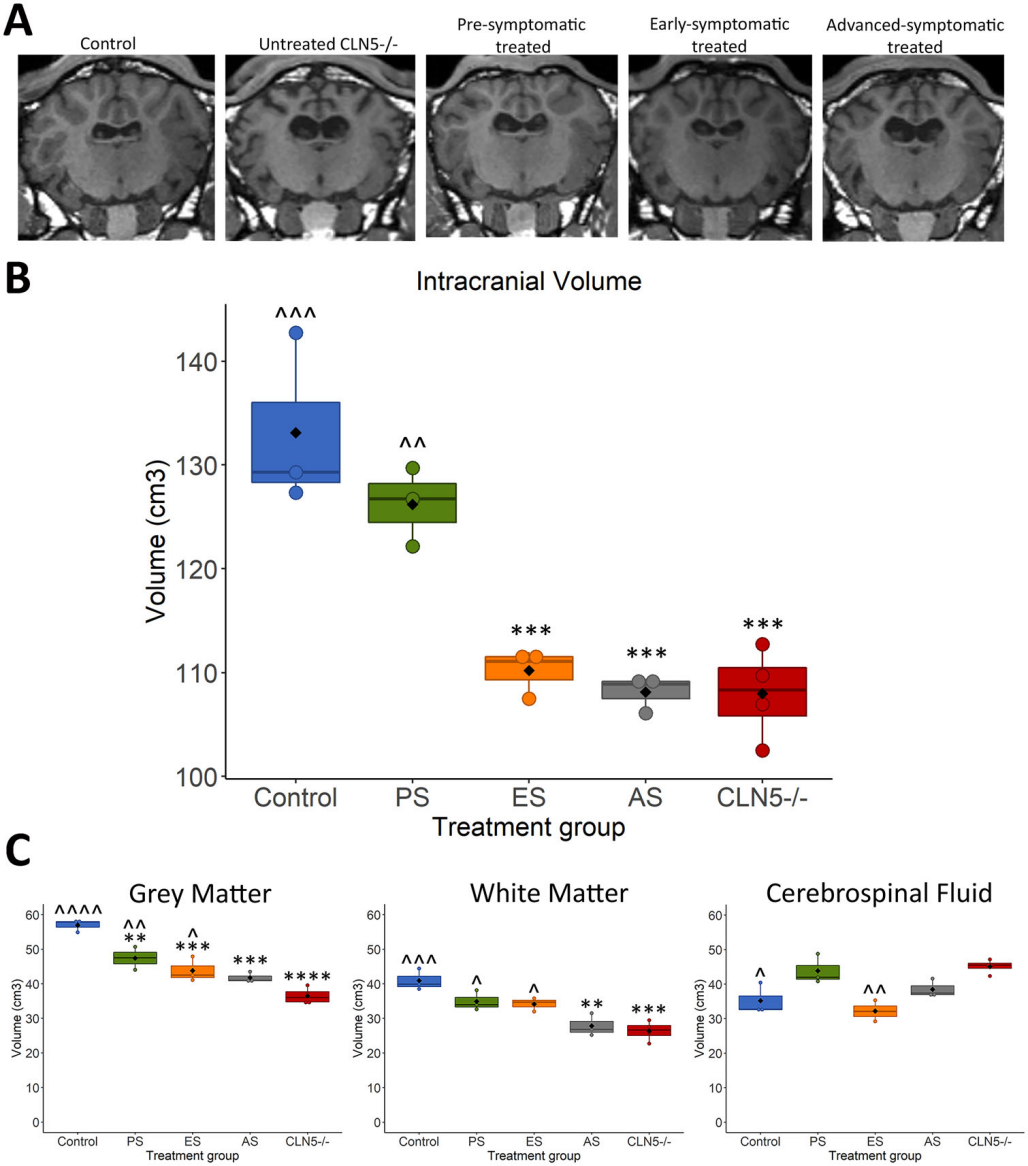
Comparison of grey matter, white matter, and cerebrospinal fluid volumes at 18 months of age following ICV/IVT treatment with scAAV9/oCLN5. (A) Representative T1‐weighted coronal MR images of the 18‐month‐old brains of a healthy control CLN5^+/−^, untreated CLN5^−/−^ sheep and ICV/IVT treated CLN5^−/−^ sheep. (B) MRI‐derived total intracranial volume in control (blue, *n* = 3), pre‐symptomatic treated (PS; green, *n* = 3), early‐symptomatic treated (ES; orange, *n* = 3), advanced‐symptomatic treated (AS; grey, *n* = 3) and untreated CLN5*^−/−^* (red, *n* = 4) sheep. (C) Grey matter, white matter, and cerebrospinal fluid volumes of each treatment group compared to control and CLN5*^−/−^* sheep. Black diamonds represent group averages, while colored circles represent volume for each individual animal. * indicates comparison to control, ^ indicates comparison to untreated CLN5^−/−^. ****p < 0.0001, ***p < 0.001, **p < 0.01, *p < 0.05.

**FIGURE 5:**
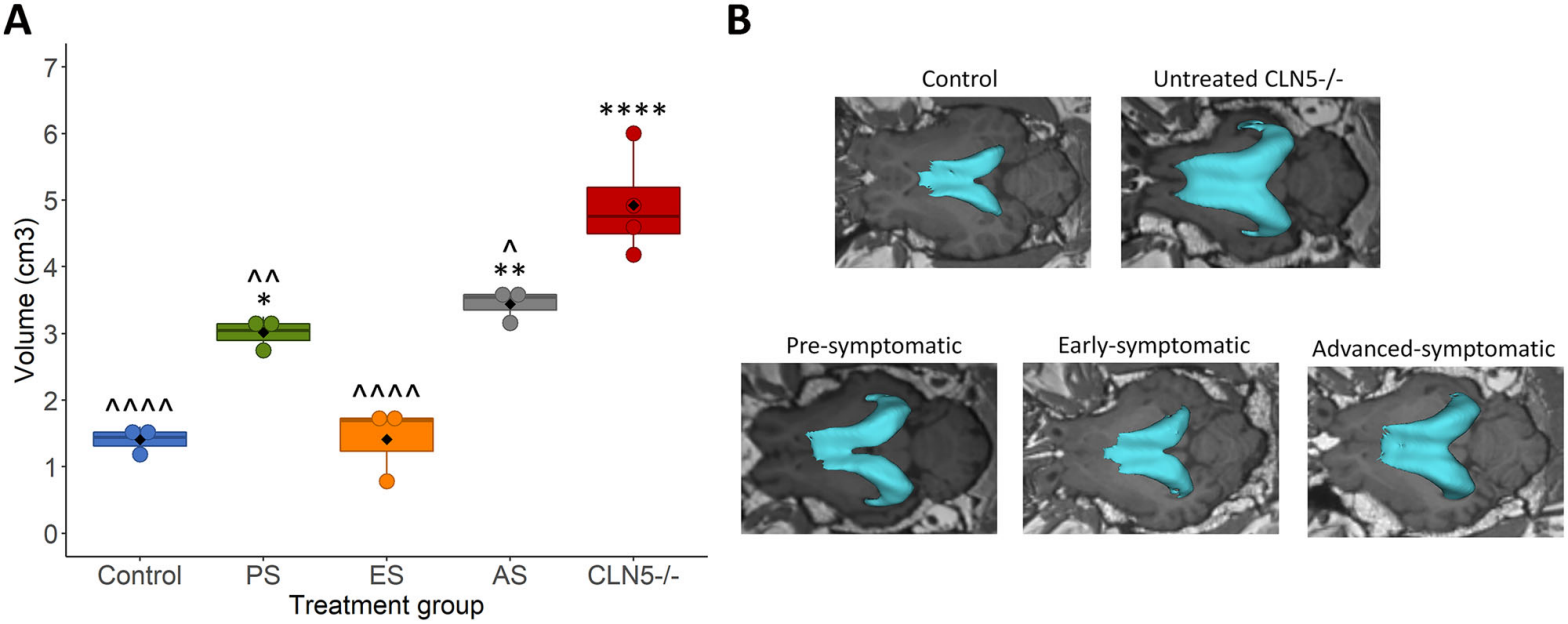
MRI‐derived lateral ventricle volumes at 18 months of age following ICV/IVT treatment with scAAV9/oCLN5. (A) MRI‐derived mean (±SEM) lateral ventricle volume of sheep that received ICV/IVT scAAV9/oCLN5 were compared to concurrent healthy control CLN5^+/−^ (blue, *n* = 3) and untreated CLN5^−/−^ sheep (red, *n* = 4) at 18 months of age. Treatment groups were pre‐symptomatic (green, *n* = 3), early symptomatic (orange, *n* = 3) or advanced symptomatic (grey, *n* = 3). (B) Representative three‐dimensional models of the cerebral lateral ventricles of ICV/IVT treated sheep overlaid onto axial T1‐weighted MR images compared to concurrent healthy control CLN5^+/−^ and untreated CLN5^−/−^ sheep. * indicates comparison to control, ^ indicates comparison to untreated CLN5^−/−^. ****p < 0.0001, *** *p* < 0.001, **p < 0.01, *p < 0.05.

**FIGURE 6:**
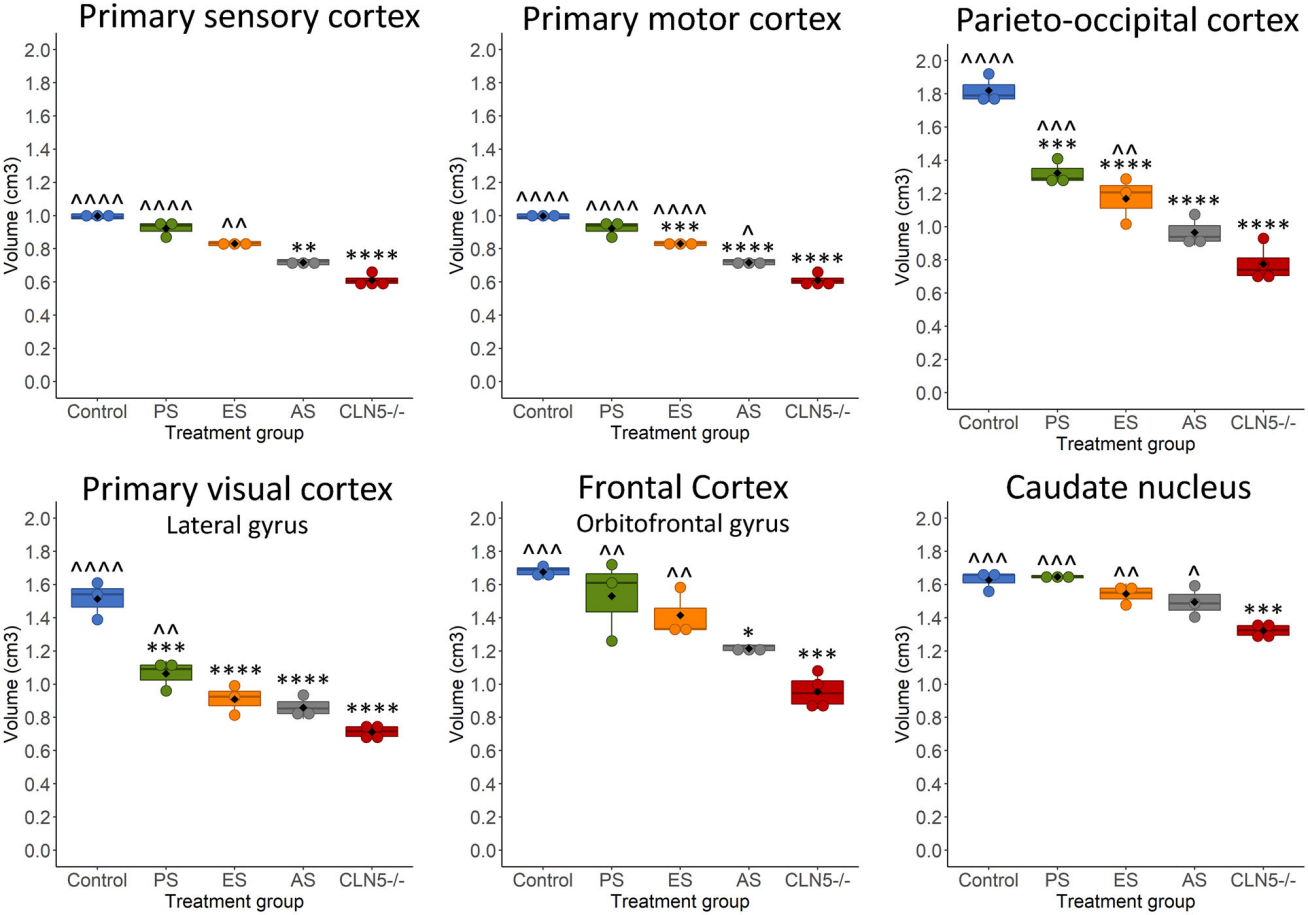
Regional MRI brain volumes at 18 months of age following ICV/IVT treatment with scAAV9/oCLN5. Mean (±SEM) regional brain volumes of sheep that received ICV/IVT scAAV9/oCLN5 were compared to concurrent healthy control CLN5^+/−^ (blue, *n* = 3) and untreated CLN5^−/−^ sheep (red, *n* = 4) at 18 months of age. Treatment groups were pre‐symptomatic (PS; green, *n* = 3), early symptomatic (ES; orange, *n* = 3) or advanced symptomatic (AS; grey, *n* = 3). * indicates comparison to control, ^ indicates comparison to untreated CLN5^−/−^. ****p < 0.0001, ***p < 0.001, **p < 0.01, *p < 0.05.

A single MRI scan was conducted on the early and advanced symptomatic high‐dose treated animals at 18 months of age. This allowed direct comparison to concurrent age‐matched pre‐symptomatic treated, control CLN5^+/−^_,_ and untreated CLN5^−/−^ brains at this time point. Comparisons of intracranial, GM, WM, and total CSF volumes were made (Figure 4), and the volumes of several regions of interest were also compared between groups (Figures 5 and 6).

At 18 months of age, pre‐symptomatic treated sheep intracranial volume was not significantly different from control and was significantly higher than untreated CLN5^−/^ *^−^* sheep (Figure 4A, B). GM volume was significantly different from both control CLN5^+/−^ and untreated CLN5^−/^ *^−^* sheep at this time point; however, WM and CSF were not significantly different from control CLN5^+/−^ sheep (Figure 4C).

Early symptomatic treated brains had intracranial volumes comparable with untreated CLN5^−/−^ brains (Figure 4A, B). GM volume was significantly different to both control CLN5^+/−^ and untreated CLN5^−/−^ brains, while WM volume was significantly higher, and CSF was significantly lower compared to untreated CLN5^−/^ *^−^* brains (Figure 4C).

Advanced symptomatic treated brains displayed ventricular enlargement and minor skull ossification (Figure 4A) and had intracranial volumes comparable to untreated CLN5^−/^ *^−^* sheep (Figure 4B). GM volume was slightly higher, and CSF volume was slightly lower compared to untreated CLN5^−/−^ sheep; however, did not reach statistical significance. WM volume was comparable to untreated CLN5^−/−^ sheep (Figure 4B).

All treatment groups showed significantly smaller lateral ventricles compared to untreated CLN5^−/−^ sheep; however, only early symptomatic treated sheep showed comparable volumes to control CLN5^+/−^ sheep (Figure 5). Of note, the average lateral ventricle volume in advanced symptomatic treated sheep was similar to 10‐month‐old untreated *CLN5^−/−^* sheep, suggesting little further ventricular enlargement from the time of injection at 9 months of age.

Several key regional volumes were also compared across the treatment and control cohorts at 18 months of age (Figure 6). These included five key cortical regions (primary visual, sensory, motor, parieto‐occipital, and frontal cortices), which were previously shown to degenerate at different rates in ovine CLN5 disease (Mitchell, Russell, et al. 2023). A clear trend was observed between age of treatment and volume. Pre‐symptomatic treated sheep had significantly greater volumes in all five cortical regions than untreated CLN5^−/−^ sheep, and a majority of these volumes were almost at healthy control volume. Cortical regions in the early symptomatic treated sheep were smaller than the pre‐symptomatic treated sheep and control CLN5^+/−^ sheep, but four of the five cortical regions (except the primary visual) were significantly larger than untreated CLN5^−/−^ sheep. Those sheep treated at an advanced symptomatic age had the smallest cortical volumes of all the treatment cohorts, but the primary motor cortex was still larger in these sheep than in age‐matched untreated CLN5^−/−^ sheep. Caudate nucleus volume was significantly reduced in untreated CLN5^−/−^ sheep, whilst treatment at any age prevented this loss (Figure 6).

To explore the consistency between MRI‐derived brain volumes and post‐mortem histology, we qualitatively compared cross‐sectional volumes from the primary motor, visual, and parieto‐occipital cortices to previously published post‐mortem cortical thickness measurements (Murray, Wellby, et al. 2023, Figure S1). MRI‐derived volumes in all three regions showed an age‐at‐treatment effect wherein pre‐symptomatic treated sheep had volumes most comparable to controls, followed by early‐symptomatic treated sheep, with advanced symptomatic sheep being more comparable to untreated CLN5*^−/−^* sheep. In contrast, post‐mortem cortical thickness showed both an age‐at‐treatment and dose effect, in that early‐symptomatic treated sheep, who received a higher ICV dose than pre‐symptomatic treated sheep, had cortical thicknesses most comparable to healthy controls in all three regions (Figure S1). However, for all regions, there was a strong positive correlation between the two measures (Figure S1C), suggesting a high degree of consistency between MRI and post‐mortem analyses. It should be noted that comparison between these two datasets is limited as the MRI‐derived volumes were obtained at 18 months of age while post‐mortem cortical thickness was obtained at 24 months.

## 4 Discussion

This study utilized structural MRI to assess the therapeutic efficacy of dual route of administration gene therapy for CLN5 Batten disease. Over 5 assessments from 5 to 18 months, sheep with CLN5 Batten disease who received moderate‐dose scAAV9/oCLN5 gene therapy pre‐symptomatically exhibited “control‐like” patterns of brain change, strikingly different from the accelerated pattern of atrophy exhibited by untreated affected sheep. A cross‐sectional study comparing global and regional brain volumes across sheep treated with scAAV9/oCLN5 at three different disease stages (pre, early, and advanced symptomatic) compared to healthy and affected controls demonstrated an effect of both age at treatment and dose on brain volumes. These studies show structural MRI to be an effective tool for assessing the therapeutic efficacy of gene therapy in a large animal model of CLN5 Batten disease.

The MRI‐derived brain volume data presented here complement the previously reported in‐life and neuropathological data from CLN5^−/^ *^−^* sheep treated with ICV/IVT scAAV9/oCLN5 (Murray, Wellby, et al. 2023). Sheep treated pre‐symptomatically showed only mild decline in clinical scores, increases in CT‐derived brain volumes over their lifetime, and low levels of neuroinflammation and lysosomal storage. Sheep treated with higher doses at early and advanced disease timepoints maintained stable clinical scores and CT‐derived brain volumes and had low levels of neuroinflammation and lysosomal storage (Murray, Wellby, et al. 2023). Together, these datasets supported the initiation of a first in‐human Phase I/II clinical trial of CLN5 gene therapy (https://clinicaltrials.gov/, NCT05228145).

### 4.1 Longitudinal MRI

The global and regional brain volume changes assessed by structural MRI in the current study show a general trend towards attenuation of atrophy in CLN5‐affected sheep that received moderate‐dose scAAV9/oCLN5 gene therapy at a pre‐symptomatic disease stage; however, the majority of measurements were not normalized to healthy control volumes. GM volume in the treated sheep did exhibit an overall decline between 5 and 18 months of age; however, the rate of change was much slower than in the untreated affected sheep. Conversely, CSF volume increased in treated sheep over the same period; however, again, the rate of increase was much slower compared to untreated affected sheep. WM and total intracranial volumes increased at the same rate in treated sheep and healthy control sheep, while showing a decline in untreated affected sheep. One treated sheep (1124/18) had relatively stable volume measurements over the course of the study. This sheep also had the best in‐life efficacy measures in her treatment group including, stable clinical scores and retained vision. Sheep 1124/18 also had the highest brain weight at post‐mortem and attenuated retinal and neuropathology (Murray, Wellby, et al. 2023).

A limitation of the longitudinal study was that sheep were only scanned until 18 months of age, whereas the terminal endpoint of the wider study was 24 months of age (Murray, Wellby, et al. 2023). In future it would be of interest to scan sheep at the endpoint of the study to marry up the clinical data and post‐mortem neuropathology findings more accurately. Nevertheless, longitudinal MRI scanning of treated sheep along with concurrent healthy and untreated affected controls has been a useful translational tool for monitoring global and regional brain volumes to inform on therapeutic efficacy in vivo, providing robust evidence for incorporation into forthcoming human trials. Using these clinically relevant outcome measures has highlighted that a moderate dose of ICV gene therapy given to CLN5‐affected sheep at a pre‐symptomatic disease stage is not sufficient to completely halt brain atrophy. Therefore, future studies may need to use higher doses at this age to optimize clinical and neuropathological outcomes.

### 4.2 Cross‐Sectional MRI

In addition to longitudinal monitoring of pre‐symptomatic treated sheep, a single timepoint scan was performed at 18 months of age on CLN5‐affected sheep who had received early and advanced symptomatic scAAV9/oCLN5 treatment (Murray, Wellby, et al. 2023). This scan allowed a cross‐sectional comparison of global and regional brain volumes in all three treatment groups along with concurrent healthy and untreated affected sheep. The results of this cross‐sectional assessment indicated that there was a combined effect of age at treatment and dose, as early and advanced symptomatic sheep were given a 10‐fold higher ICV dose compared to pre‐symptomatic treated sheep. Despite pre‐symptomatic treated sheep having similar intracranial volumes to healthy controls at 18 months of age, a greater proportion of the total volume in pre‐symptomatic treated sheep was attributed to CSF volume compared to controls. Early symptomatic treatment with a higher dose appeared to be the most efficacious at 18 months of age. GM and WM volumes were significantly higher than in untreated affected sheep of the same age. In addition, the lateral ventricle volumes and total CSF volumes were most comparable to healthy control sheep in the early symptomatic treated group.

A cross‐sectional study to assess therapeutic efficacy is only useful if there is robust natural history data to compare to at that particular point in time. In the current study such natural history data was available; however, in the absence of this, a single MRI scan may not be useful for assessing disease stage or therapeutic efficacy. Therefore, while the cross‐sectional study has been informative in this preclinical context, it may not be as useful in a clinical situation, which is much more suited to within‐subject longitudinal tracking.

### 4.3 MRI is a Clinically Relevant Tool to Assess Therapeutic Efficacy in Pre‐Clinical Models

MRI is a widely used imaging modality to assess disease stage and therapeutic efficacy in both preclinical and clinical studies. For NCL, clinical use of MRI has primarily been restricted to case studies to confirm diagnosis or disease stage or small longitudinal studies assessing brain changes over disease course (Biswas et al. 2020; Dyke et al. 2016; Jadav et al. 2014; Löbel et al. 2016; Luo et al. 2020). Similarly, MRIs in preclinical models of NCL have primarily been performed as case studies or natural history studies to establish timelines of disease progression (Eaton et al. 2019; Meiman et al. 2022; Munasinghe et al. 2012; Sawiak et al. 2015; Seo et al. 2022; White et al. 2018). Very few studies have utilized MRI to systematically track therapeutic efficacy in NCL. However, there are several studies highlighting the relevance and suitability of MRI as an efficacy tool in preclinical studies of other types of lysosomal storage diseases. In dogs with mucopolysaccharidosis type I (MPSI), MRI was used to assess the efficacy of enzyme replacement therapy. A total of 32 dogs were scanned between 12 and 30 months of age, and results showed that although treated dogs did display some cortical atrophy and ventricular enlargement, this was less pronounced than in untreated dogs (Vite et al. 2013). In a sheep model of Tay–Sachs disease, MRI was performed at a humane endpoint in affected sheep and sheep that had received ICV and intrathalamic gene therapy and compared to healthy control sheep scanned between 9 and 12 months of age (Gray‐Edwards et al. 2018). Results showed a normalization of grey to WM intensities, which are typically abnormal in affected sheep; however, cortical atrophy was apparent in treated sheep at humane endpoint. A similar study was performed in cats with GM1 gangliosidosis who received ICV and intrathalamic gene therapy, in which MRI scans revealed that treatment ameliorated intensity changes and preserved brain architecture (Gray‐Edwards et al. 2020).

## 5 Conclusions

MRI is a clinically relevant outcome measure used here to complement a wider preclinical study assessing a novel dual‐route administration gene therapy for CLN5 Batten disease. The current study utilized MRI to assess the efficacy of AAV9‐mediated gene therapy in sheep with naturally occurring CLN5 Batten disease. The longitudinal study showed that treatment ameliorated global brain atrophy, ventricular enlargement, and atrophy of key brain regions such as the sensory, motor, and visual cortices. The cross‐sectional study showed that age‐at‐treatment and dose‐response effects were evident at 18 months of age in both global and regional brain volumes. Overall, these studies highlighted the usefulness of MRI both in monitoring treatment efficacy over time and, when sufficient natural history data exists, providing a picture of efficacy at a single time point.

## Author Contributions

**Samantha J. Murray**: conceptualization, methodology, data curation, formal analysis, investigation, writing – original draft, writing – review and editing. **Mustafa M. Almuqbel**: methodology, writing – review and editing. **Simon A. Felton**: methodology, writing – review and editing. **Nickolas J. Palmer**: methodology, writing – review and editing. **Ashley R. Deane**: methodology, formal analysis, writing – review and editing. **Daniel J. Myall**: methodology, data curation, writing – review and editing. **Reza Shoorangiz**: methodology, writing – review and editing. **Arsène Ella**: methodology. **Matthieu Keller**: methodology, writing – review and editing, resources. **David N. Palmer**: writing – review and editing, funding acquisition, project administration. **Tracy R Melzer**: conceptualization, methodology, data curation, supervision, formal analysis, writing – review and editing, resources. **Nadia L. Mitchell**: conceptualization, methodology, investigation, formal analysis, supervision, project administration, writing – review and editing, writing – original draft.

## Conflicts of Interest

The authors declare no conflicts of interest.

### Peer Review

The peer review history for this article is available at https://publons.com/publon/10.1002/brb3.70431.

## Supporting information



Supplementary Figure 1. Comparison of MRI‐derived cortical volumes to terminal cortical histopathology.

## Data Availability

The data that support the findings of this study are available from the corresponding author upon reasonable request.
