# Supplementary material for: Magnetic Resonance Imaging as a Readout of CLN5 Gene Therapy Efficacy in Sheep
Source: Brain Behav. 2025 Apr 3;15(4):e70431. doi: 10.1002/brb3.70431 (PMC11968780; doi:10.1002/brb3.70431)
Supplement: Supplementary file 1 — Supplementary Figure 1. Comparison of MRI‐derived cortical volumes to terminal cortical histopathology. [file BRB3-15-e70431-s001.pdf]

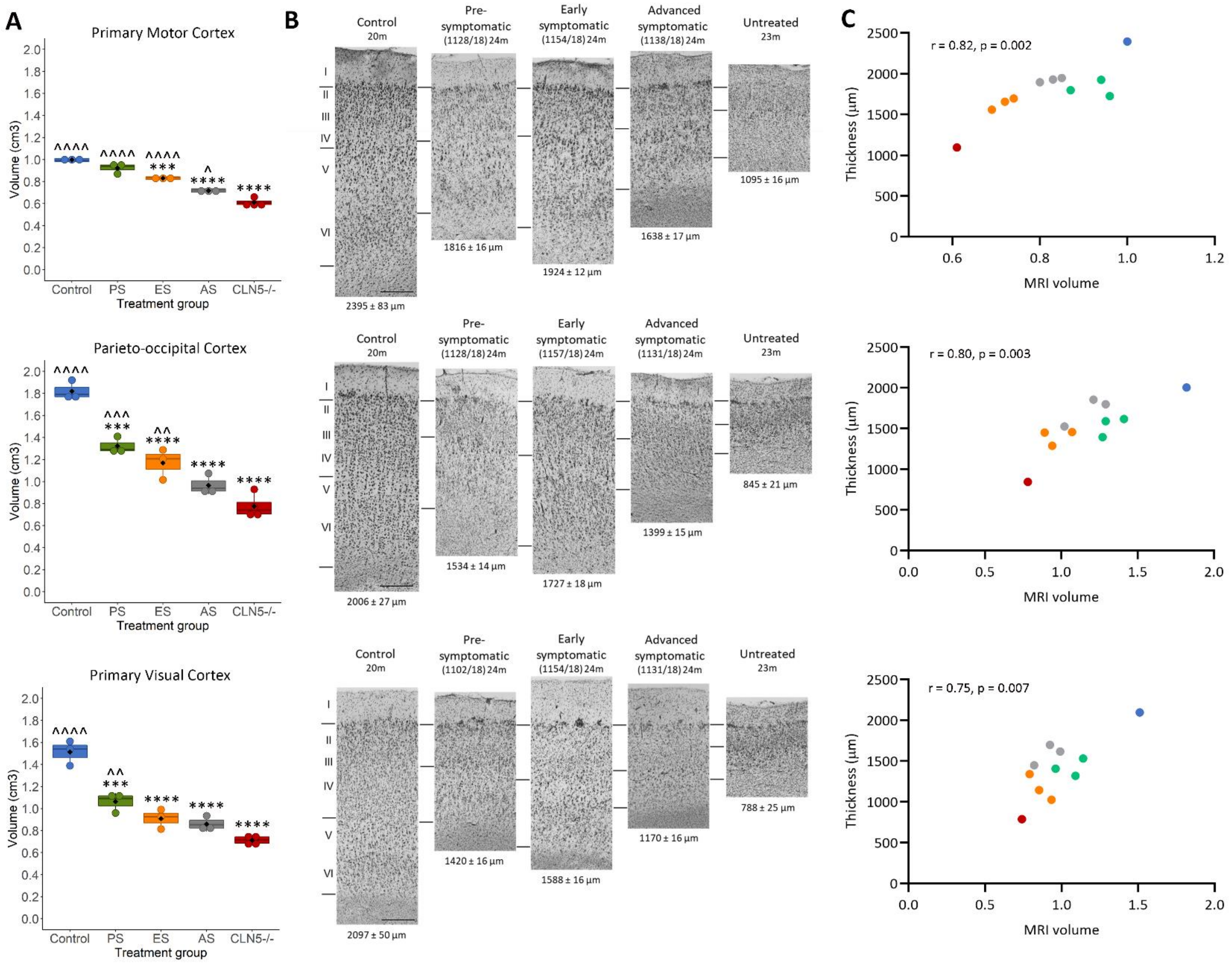

Supplementary Figure 1. Comparison of MRI-derived cortical volumes to terminal cortical histopathology. (A) Longitudinal MRI-derived volume changes in the primary motor cortex (M1), parieto-occipital cortex (POC), and primary visual cortex (V1) in healthy control (blue), pre-symptomatic ICV/IVT treated (green), early symptomatic ICV/IVT treated (orange), advanced symptomatic treated (grey), and untreated CLN5-/- affected (red) sheep. Graphs extracted from Figure 6. * indicates comparison to control, ^ indicates comparison to untreated CLN5$^{-/-}$. **** p<0.0001, *** p<0.001, ** p<0.01, *p<0.05. **(B)** Representative images of Nissl-stained tissue from M1, POC, and V1 demonstrating the cortical thickness at 24 months of age (terminal endpoint of the study). Scale bar represents 200µm. Mean (± SEM) cortical thicknesses for each group are noted beneath the images. **(C)** Linear relationship between MRI-derived volumes and post-mortem cortical thickness and corresponding Pearson correlation co-efficient (r) and p-value.
